# Interactions between* CYP11B2* Promoter Methylation and Smoking Increase Risk of Essential Hypertension

**DOI:** 10.1155/2016/1454186

**Published:** 2016-12-19

**Authors:** Tianlun Gu, Shuqi Mao, Rui Fan, Fade Zhong, Fubao Zhu, Lingmei Hao, Lina Zhang, Fengying Yin

**Affiliations:** ^1^Department of Preventative Medicine, Zhejiang Provincial Key Laboratory of Pathological and Physiological Technology, Medicine School of Ningbo University, 818 Fenghua Road, Ningbo, Zhejiang Province 315211, China; ^2^Department of Medical Quality, Ningbo Medical Center Lihuili Eastern Hospital, Ningbo, Zhejiang 315211, China; ^3^Ningbo Central Blood Station, Ningbo, Zhejiang 315211, China; ^4^Health and Family Planning Bureau of Zhenhai District, Ningbo, Zhejiang, China; ^5^Clinical Laboratory, The Seventh Hospital of Ningbo, Ningbo, Zhejiang 315211, China; ^6^Clinical Laboratory, The First Hospital of Ningbo, Zhejiang, China

## Abstract

Aldosterone synthase (CYP11B2) is closely linked to essential hypertension (EH). However, it remains unclear whether the methylation of the* CYP11B2* promoter is involved in the development of EH in humans. Our study is aimed at evaluating the contribution of* CYP11B2* promoter methylation to the risk of EH. Methylation levels were measured using pyrosequencing technology in 192 participants in a hospital-based case-control study. Logistic regression and multiple linear regression analyses were utilized to adjust for confounding factors and the GMDR method was applied to investigate high-order gene-environment interactions. Although no significant result was observed linking the four analyzed CpG sites to EH, GMDR detected significant interactions among CpG1, CpG3, CpG4, and smoking correlated with an increased risk of EH (OR = 4.62, adjusted *P* = 0.011). In addition, CpG2 (adjusted *P* = 0.013) and CpG3 (adjusted *P* = 0.039) methylation was significantly lower in healthy males than in healthy females. Likewise, after adjusting for confounding factors, CpG2 methylation (adjusted *P* = 0.007) still showed significant gender-specific differences among the participants of the study. CpG1 (*P* = 0.009) site was significantly positively correlated with age, and CpG3 (*P* = 0.007) and CpG4 (*P* = 0.006) were both inversely linked to smoking. Our findings suggest that gene-environment interactions are associated with the pathogenesis and progression of EH.

## 1. Introduction

Essential hypertension (EH), a complex chronic noncommunicable disease, is a major risk factor for cardiovascular disease (CVD) and mortality, determined by multiple genetic and environmental factors. Annually, approximately 9.4 million deaths are attributed to high blood pressure as one of three leading risk factors for global disease burden (GDB) [1, 2]. Although the mechanisms underlying the blood pressure changes in EH remain to be fully elucidated, the renin-angiotensin-aldosterone system (RAAS) has been documented to play crucial roles.

Aldosterone synthase (CYP11B2), an important member of RAAS, is a key enzyme for the synthesis of aldosterone and the* CYP11B2* gene has been listed as one of the candidate genes of EH. Previous evidence from Stella et al. suggested that aldosterone synthase -344C/T polymorphism was positively correlated with left ventricular (LV) mass and thickness in EH [3, 4]. The* CYP11B2* gene -344C/T polymorphism is also associated with atrial fibrillation (AF) [5, 6]. Another recent study also identified the variants of* CYP11B2* associated with increased risk of EH [7]. Although the influences of genetic factors on blood pressure (BP) variation in humans are estimated to be responsible for 30% to 50% [8], an alteration of DNA without sequence changes, named epigenetics, likely plays crucial roles as well.

Promoter methylation of genes susceptible to EH has been investigated in numerous recent studies. Hypomethylation of* AGTR1b* as a result of protein-poor maternal diet in gestating fetuses, which could lead to overexpression of the gene in the adrenal glands, has been implicated in the development of hypertension during adulthood in rats [9, 10]. Similarly, hypomethylation of the* AGTR1* promoter was reported to be associated with the risk of EH in humans [11]. Rangel et al. also observed that discrete methylation changes in the* ACE* promoter had an effect on systolic blood pressure (SBP) and angiotensin I converting enzyme (ACE) activity levels [12]. Based on current evidence, it is clear that aberrant methylation of genes in RAAS is closely related to EH.

As EH is a complex and multifactorial disease, gene-environment interactions contribute significantly to the onset and progression of EH. However, due to the “curse of dimensionality,” structuring models based on traditional statistical methods has already been deemed unfit for investigating potential or undetectable interactions. Recently, a generalized multifactor dimensionality reduction (GMDR) approach emerged as a promising tool and has been used for detecting gene-gene and gene-environment interactions in either dichotomous or continuous phenotypes, as well as permitting adjustment for discrete and quantitative covariates and enhancing prediction accuracy [13, 14]. The GMDR method was successfully employed to deduce the interaction between* ADD1* methylation and alcohol consumption leading to increased risk of EH [15].

To date, however, the association between* CYP11B2* methylation and EH risk is unclear. Thus, the goal of the current study is to explore whether the aberrant methylation of* CYP11B2* is linked to EH risk and further explore the roles of gene-environment interactions in EH risk. In addition, we intend to detect the association among age, gender, clinical variables, and DNA methylation, which are demonstrated in previous studies.

## 2. Materials and Methods

### 2.1. Ethics Statement

This was a hospital-based case-control study. The study protocol conformed to the ethical guidelines of the 1975 Declaration of Helsinki and was approved by the Ethics Committee of Ningbo Seventh Hospital. Written informed consent was obtained from all the participating subjects.

### 2.2. Study Population

A total of 192 individuals, 96 cases with and the other half without EH, were recruited at the Ningbo Seventh Hospital for health examination in Ningbo City, Zhejiang, China. Patients were defined as hypertensive based on systolic blood pressure (SBP) ≥ 140 mmHg or diastolic blood pressure (DBP) ≥ 90 mmHg, in accordance with the 2013 European Society of Hypertension and European Society of Cardiology Guidelines [16]. In addition, all hypertensive participants were incident cases receiving no therapy for hypertension regardless of drug, physical status, and nutrition. Normotensive controls, with SBP < 120 mmHg and DBP < 80 mmHg, had no history of diabetes mellitus (DM), secondary hypertension, myocardial infarction, stroke, renal failure, drug abuse, or other serious diseases and no family history of hypertension in first-degree relatives and were well matched in age (±3 years) and sex within the case group. All the participants, 30 to 75 years old, were sampled from the Han nationality residing in Ningbo for at least three generations. Blood pressure was measured twice at an interval of 10 min in the supine position and the mean value of the two readings was recorded by diverse trained technicians using a calibrated mercury sphygmomanometer with an adult-sized cuff according to standard protocols of the American Heart Association [17, 18]. Following successive 12 h fast overnight, blood samples were drawn from the antecubital vein, using vacutainer tubes containing EDTA, by licensed practical nurses and stored at −80°C for DNA extraction.

### 2.3. Biochemical Analyses

Routine blood tests including total cholesterol, triglyceride, uric acid, low-density lipoprotein (LDL), high-density lipoprotein (HDL), homocysteine (Hcy), and glucose were measured enzymatically, using an AU2700 automatic analyzer (Olympus, Tokyo, Japan). Genomic DNA was extracted from peripheral blood samples using a Lab-Acid 820 nucleic acid extraction analyzer (Zeesan Biotech, Xiamen, China), and the DNA concentration was detected using a NanoDrop 2000 ultra-micro nucleic acid ultraviolet tester (Thermo Fisher Scientific, Wilmington, DE, USA).

### 2.4. Pyrosequencing Array

Firstly, we positioned CpG island (CGI) using MethPrimer (http://www.urogene.org/cgi-bin/methprimer/methprimer.cgi) [19]. In addition, for the CpG sites of the promoter region of our interest, PCR primers were designed based on general principles and suggestion of primer design [20] and the scores were automatically outputted by the PyroMark Assay Design v2.0.1.15 (Qiagen). To measure DNA methylation levels, we employed pyrosequencing, a novel widely used sequencing-by-synthesis method, in our study. The explicit operational program was employed and described in our previous research. Briefly, unmethylated cytosine residues of the target sequences of the candidate gene are first converted to thymine, using sodium bisulfite (EpiTect Bisulfite Kit, Qiagen, Hilden, Germany). The converted DNA is then amplified by polymerase chain reaction (PCR) using Mastercycler Nexus Gradient (Eppendorf, Hamburg, Germany), followed by “sequencing by synthesis” of the target (PyroMark Gold Q96, Qiagen) [21, 22]. The PCR reaction mixture consisted of 12 *μ*L ZymoTaq™ Premix (Zymo Research Corporation, Irvine, CA, USA), 8 *μ*L DNase/RNase-free water, 2 *μ*L converted DNA, and 1.5 *μ*L of each of the forward and reverse primers. The target was amplified by the forward primer 5′-GTGGTGGTGGTATATGTTT-3′ and the reverse primer 5′-ATCCCCCAAACTAAAATACA-3′, and the primer 5′-TTAGTTATTTGGGAGG-3′ was used to detect the target sequence. The reaction cycle comprised initial denaturation at 95°C for 10 min, followed by amplification at 95°C for 30 sec, 46.8°C for 40 sec, and 72°C for 50 sec over 45 cycles, followed by final extension at 72°C for 7 min.

### 2.5. Statistical Analyses

Data analysis was performed on PASW Statistics 18.0 (SPSS, Inc., Somers, NY, USA). Results are presented as the mean ± SD or number (percentages) of patients. Continuous variables such as DNA methylation, age, body mass index (BMI), total cholesterol (TC), triglycerides (TG), glucose, blood uric acid (BUA), high- and low-density lipoprotein, and homocysteine (Hcy) were compared using independent samples *t*-test or nonparametric test. Pearson chi-square or Fisher's exact test was used to evaluate the correlation between EH and categorical variables such as gender, tobacco usage, and alcohol consumption. Pearson correlation analysis was used to investigate interactions among the four CpG sites in the* CYP11B2* promoter. Logistic regression and multiple linear regression were performed with the objective of detecting true association after adjustment for confounders. GMDR was used to investigate high-order interactions among diverse variables.

## 3. Results

### 3.1. Baseline Characteristics of the Study Participants

A total of 192 participants (mean age: 57.0 ± 8.5 years; 60.4% women) including 96 patients with EH and 96 gender- and age-matched (±3 years) healthy controls were enrolled, and their baseline characteristics are presented in Table 1. No significant differences in gender and age were detected between EH patients and healthy controls (*P* > 0.05), demonstrating the well-restricted nature of the study population for gender and age.

**Figure 1:**
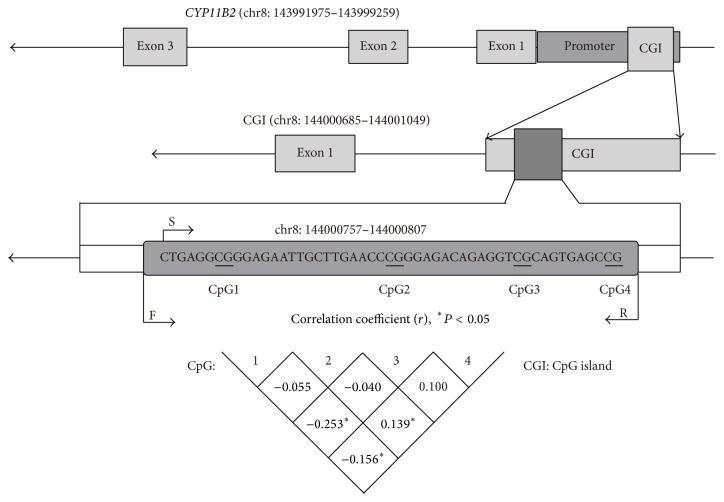
The four analyzed CpG sites in* CYP11B2*.

CpG island (CGI) was identified in the promoter of the* CYP11B2* gene by using MethPrimer (http://www.urogene.org/cgi-bin/methprimer/methprimer.cgi) [19]. Subsequently, a target fragment containing four CpG dinucleotides in the above target island (chr8: 144000685–144001049) was selected (Figure 1). The correlation among these four CpG sites is presented in Figure 1 (*r* < 0.5).

### 3.2. Interactions between Promoter Methylation and Environmental Factors to EH

According to our results, EH was not significantly associated with methylation levels of all four CpG sites after adjusting for age, gender, BUA, smoking, drinking, BMI, TG, HDL, LDL, and Hcy. Due to the complex and multifactorial nature of EH, the effect of a single or several sites cannot sufficiently explain the pathogenesis of EH. Subsequently, interactions between methylation of CpG sites and environmental factors including smoking and alcohol use were studied with GMDR. The best models at various orders are summarized in Table 2. The four-factor interaction model of tobacco usage and DNA methylation (CpG1, CpG3, and CpG4) was the best model, along with the best training balanced accuracy (0.68), testing balanced accuracy (0.60), and cross-validation consistency (10/10). The consistency of the model across 10-fold cross-validation training sets is that how many times the same GMDR model is identified in all the possible training sets. Among this set of the best significant models (*P* < 0.05), we pick the model that has the maximum prediction accuracy with the least error and/or maximum cross-validation consistency. The model's adjusted *P* value was 0.011 after the sign test, and odds ratio (OR) was 4.62 with 95% confidence interval (1.49, 14.34).

### 3.3. Analysis of Clinical Variables and EH

**Figure 2:**
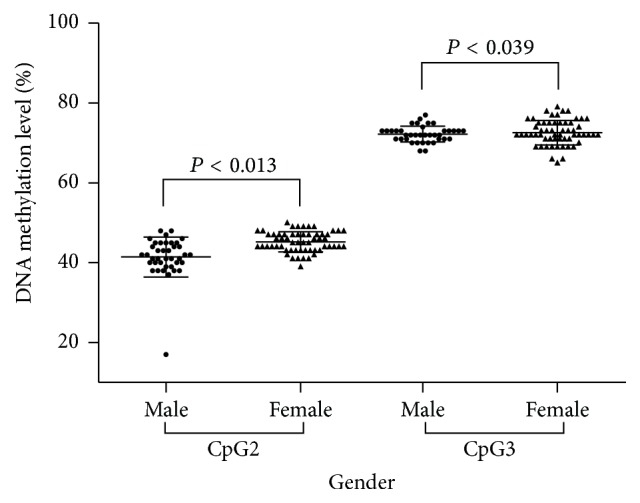
Significant difference of promoter methylation of* CYP11B2* between male and female in healthy control.

Methylation of CpG2 (adjusted *P* = 0.013) and CpG3 (adjusted *P* = 0.039) was significantly lower in healthy males than in healthy females after adjusting for confounding factors (Table 3 and Figure 2). However, there were no significant differences between EH in males and females for the four CpG sites' methylation (Table 4). After adjusting for confounding factors, CpG2 methylation (adjusted *P* = 0.007) still showed a significant gender-specific difference among all the participants (Table 5). As listed in Table 1, significant differences between essential hypertensive and healthy participants were also detected in ALP (*P* = 0.002), GGT (*P* = 0.006), BMI (*P* < 0.001), TG (*P* = 0.032), HDL (*P* < 0.001), and BUA (*P* = 0.025). Therefore, multiple linear regression was used for testing whether these clinical variables were associated with methylation of the* CYP11B2* promoter in different dimensionalities. As a result, in healthy controls, CpG1 (*β* = 0.104, *P* = 0.009) site was significantly positively correlated with age, and CpG3 (*β* = −2.485, *P* = 0.007) and CpG4 (*β* = −5.089, *P* = 0.006) were both inversely linked to smoking (Table 3). However, in EH patients, just CpG1 (*β* = 0.117, *P* = 0.001) site was significantly positively correlated with age (Table 4). And, in the whole study population, CpG1 (*β* = 0.112, *P* < 0.001) site was significant positively correlated with age and CpG4 (*β* = −2.664, *P* = 0.034) site was negatively linked to smoking (Table 5).

## 4. Discussion

Previous studies demonstrated that the polymorphic variation of* CYP11B2*, the gene encoding aldosterone synthase, was associated with increased aldosterone metabolite excretion and hypertension [23–27]. In the current study, we hypothesized that aberrant methylation of the* CYP11B2* promoter might also account for EH risk. Although no significant link was observed between the four analyzed CpG sites and EH, the interactions between* CYP11B2* promoter methylation (CpG1, CpG3, and CpG4) and smoking were significantly correlated with the risk of EH. In addition, we found that methylation of CpG2 and CpG3 was significantly different between males and females in healthy controls. CpG2 methylation exhibited gender-specific differences among all the participants, and CpG1 methylation was significantly positively linked to age, while CpG3 and CpG4 methylation was negatively correlated with smoking. Our results broaden the picture of the pathogenesis of EH.

In general, promoter hypermethylation suppresses transcription, but hypomethylation activates transcription [28]. Therefore, if promoter methylation of the gene encoding key enzymes involved in the pathogenesis of EH was negatively correlated with the incidence of EH, its hypomethylation would increase the prevalence risk of EH. Indeed, promoter hypomethylation augments expression of AGTR1, which increases the risk of EH [11]. One study has demonstrated, based on integrated analysis of genome-wide methylation and gene expression data in the adrenal cortex tissue samples, that the* CYP11B2* gene is differentially methylated and expressed through an epigenetic mechanism [29]. In the current study, we did not find any significant results between the four tested CpG methylation instances of* CYP11B2* promoter and EH, even though the statistical power of our study was 0.931. Epigenetic changes, however, could interplay with or be mediated through a multitude of environmental and dietary factors. With the GMDR method, we structured gene-environment interaction models and detected a crucially significant four-order synergistic effect among CpG1, CpG3, and CpG4 methylation and smoking (*P* = 0.011), contributing strongly to the risk of EH. Previous studies consistently reported that some factors had no main effects whereas the interactions played key roles. According to the evidence presented by Taylor et al., two SNPs were detected providing support for powerful genetic interactions with cigarette smoking associated with SBP in African Americans from genome-wide studies, but no main genetic effect linked to SBP was detected with only these two SNPs in the model [30]. Smoking is a major environmental risk factor of EH, and previous studies reported that interactions between environmental factors and epigenetic phenomena may be synergistic [31]. Indeed, the present study found that individuals with hypomethylated CpG3 and CpG4 sites had 4.62-fold increased risk of EH when coupled with smoking, further documenting the interactions between DNA methylation and environmental factors, which could influence the development of EH. Nevertheless, the concrete biological mechanism of the above interactions remains unclear, and further studies are required.

In our research, we found significantly lower CpG2 and CpG3 methylation levels in males compared to females in healthy controls. After adjusting for confounding factors, CpG2 methylation still showed a significant gender-specific difference among the participants. Such sex-related differences are common and widespread in the incidence of CVD [32, 33]. Steroid sex hormones have been postulated as the primary contributor to these sex-specific differences. Estrogen is widely accepted as a positive effector, providing significant protection against the development of CVD, but these benefits fade away following menopause [34], while testosterone is thought to be harmful or detrimental to heart function. Moreover, DNA methylation profiles show that the dynamic regulatory elements are responsive to environmental stimuli, such as tobacco usage, alcohol consumption, sedentariness, high-sodium diet, age, and other silent inimical-to-health factors [35–37], altering the DNA methylation intensities of EH risk genes and causing EH over time. Indeed, our results also revealed that smoking interplayed with DNA methylation. Meanwhile, environmental factors listed above can be hazardous to people's health leading to the development of chronic diseases. Since lifestyle or behavior factors such as tobacco usage, alcohol consumption, and lack of physical exercise are too inveterately formed to change for people, and, of difference between females and males, our results of sex-related* CYP11B2* methylation discrepancy may reveal in advance that these acquired nonhereditary risk factors could influence the genetic variants via diverse enigmatic mechanisms. Earlier studies have also reported age-related changes in DNA methylation [38, 39]. We also found that CpG1 was negatively correlated with age, but the mechanism remains unclear, which requires further study.

In summary, our study detects the interactions between the* CYP11B2* promoter methylation (CpG1, CpG3, and CpG4) and smoking, which may increase the risk of EH. Further, age, gender, and smoking are likely to influence* CYP11B2* methylation levels. These findings can help us further the understanding of the pathogenesis of EH.

## Figures and Tables

**Table 1:** Baseline characteristics of the study participants (*N* = 192).

Characteristic	EH	Non-EH	*t*/*χ* ^2^	*P*
Age (years)	56.72 ± 8.71	56.32 ± 8.23	0.324	0.747
Gender	38/58	38/58	0	1.000
ALP (IU/I)	70.10 ± 9.18	74.89 ± 11.85	3.125	**0.002**
GGT (U/L)	44.10 ± 17.29	36.11 ± 22.01	−2.796	**0.006**
BUN (mmol/L)	5.04 ± 1.11	4.96 ± 1.07	−0.455	0.650
Scr (*μ*mol/L)	83.46 ± 11.04	82.68 ± 12.28	−0.463	0.644
BUA (*μ*mol/L)	325.75 ± 82.63	300.32 ± 73.15	−2.258	**0.025**
TG (mmol/L)	1.43 ± 0.72	1.21 ± 0.68	−2.165	**0.032**
HDL (mmol/L)	2.07 ± 5.58	7.99 ± 6.32	6.890	<**0.001**
LDL (mmol/L)	3.31 ± 0.68	3.21 ± 0.87	0.924	0.357
Hcy (*μ*mol/L)	12.69 ± 2.27	11.94 ± 5.23	−1.286	0.200
BMI (kg/m^2^)	23.57 ± 3.10	22.16 ± 2.30	−3.580	<**0.001**
Smoking (Y/N)	27/69	17/79	2.948	0.086
Drinking (Y/N)	40/56	31/65	1.810	0.178
CpG1 methylation (%)	4.38 ± 2.94	4.09 ± 2.98	0.653	0.983^a^
CpG2 methylation (%)	44.19 ± 3.08	43.72 ± 4.15	1.039	0.279^a^
CpG3 methylation (%)	73.12 ± 3.20	72.46 ± 2.71	1.606	0.461^a^
CpG4 methylation (%)	62.80 ± 5.301	63.05 ± 5.28	0.374	0.798^a^

^a^ *P* was adjusted by logistic regression for age, gender, BUA, smoking, drinking, BMI, TG, HDL, LDL, and Hcy. BMI: body mass index; ALP: alkaline phosphatase; TG: triglycerides; HDL: high-density lipoprotein; LDL: low-density lipoprotein; Hcy: homocysteine.

**Table 2:** GMDR models of high-order gene-environment interaction in *CYP11B2 *promoter on EH risk.

Model	Training balanced accuracy	Testing balanced accuracy	Sign test (*P*)	Cross-validation consistency
CpG2	0.56	0.45	3 (0.945)	6/10
CpG3, smoking	0.61	0.52	7 (0.172)	6/10
CpG1, CpG3, drinking	0.64	0.49	5 (0.623)	5/10
CpG1, CpG3, CpG4, smoking	0.68	0.60	9 (**0.011**)	10/10
CpG1, CpG3, CpG4, smoking, drinking	0.72	0.53	7 (0.172)	8/10
CpG1, CpG2, CpG3, CpG4, smoking, drinking	0.75	0.51	6 (0.377)	10/10

*P* value was adjusted for age, gender, BMI, triglycerides, HDL, BUA, and Hcy using logistic regression in GMDR analysis.

GMDR: generalized multifactor dimensionality reduction; BMI: body mass index; TG: triglycerides; HDL: high-density lipoprotein; BUA: blood uric acid; Hcy: homocysteine.

**Table 3:** Association of promoter methylation with environmental factors in control.

Variable	Gender			Age		Smoking	
	Men	Women	*P*	*β* (95% CI)	^*∗*^ *P*	*β* (95% CI)	^*∗*^ *P*
CpG1	3.45 ± 2.58	4.52 ± 3.16	0.983	0.104 (0.014, 0.194)	**0.009**	1.949 (−0.362, 4.260)	0.055
CpG2	41.42 ± 5.07	45.22 ± 2.51	**0.013**	−0.003 (−0.123, 0.117)	0.960	−2.215 (−5.250, 0.820)	0.096
CpG3	72.24 ± 1.99	72.60 ± 3.10	**0.039**	−0.047 (−0.128, 0.034)	0.148	−2.485 (−4.551, −0.419)	**0.007**
CpG4	62.13 ± 7.68	63.66 ± 2.71	0.082	−0.051 (−0.215, 0.113)	0.477	−5.089 (−9.254, −0.924)	**0.006**

*P* was adjusted by logistic regression for age, BUA, smoking, drinking, BMI, ALP, TG, HDL, LDL, and Hcy. ^*∗*^ *P* was calculated by multiple linear regression model including gender, age, BUA, smoking, drinking, BMI, ALP, TG, HDL, LDL, and Hcy. BMI: body mass index; ALP: alkaline phosphatase; TG: triglycerides; HDL: high-density lipoprotein; LDL: low-density lipoprotein; Hcy: homocysteine.

**Table 4:** Association of promoter methylation with environmental factors in EH patients.

Variable	Gender			Age		Smoking	
	Men	Women	*P*	*β* (95% CI)	^*∗*^ *P*	*β* (95% CI)	^*∗*^ *P*
CpG1	3.55 ± 2.47	4.91 ± 3.12	0.997	0.117 (0.049, 0.185)	**0.001**	−0.318 (−2.390, 1.754)	0.761
CpG2	42.58 ± 3.03	45.24 ± 2.64	0.997	−0.005 (−0.123, 0.117)	0.899	0.374 (−1.795, 2.544)	0.732
CpG3	73.05 ± 3.27	73.17 ± 3.17	1.000	−0.037 (−0.128, 0.034)	0.356	0.533 (−1.871, 2.938)	0.660
CpG4	61.42 ± 7.38	63.71 ± 3.07	0.992	−0.011 (−0.144, 0.122)	0.868	−1.804 (−5.850, 2.242)	0.378

*P* was adjusted by logistic regression for age, BUA, smoking, drinking, BMI, ALP, TG, HDL, LDL, and Hcy. ^*∗*^ *P* was calculated by multiple linear regression model including gender, age, BUA, smoking, drinking, BMI, ALP, TG, HDL, LDL, and Hcy. BMI: body mass index; ALP: alkaline phosphatase; TG: triglycerides; HDL: high-density lipoprotein; LDL: low-density lipoprotein; Hcy: homocysteine.

**Table 5:** Association of promoter methylation with environmental factors in whole participants.

Variable	Gender			Age		Smoking	
	Men	Women	*P*	*β* (95% CI)	^*∗*^ *P*	*β* (95% CI)	^*∗*^ *P*
CpG1	3.50 ± 2.51	4.72 ± 3.13	0.181	0.112 (0.062, 0.162)	**<0.001**	0.742 (−0.640, 2.123)	0.291
CpG2	42.00 ± 4.19	45.23 ± 2.57	**0.007**	0.001 (−0.059, 0.060)	0.983	−0.659 (−2.314, 0.996)	0.433
CpG3	72.64 ± 2.72	72.89 ± 3.13	0.405	−0.042 (−0.095, 0.010)	0.115	−0.401 (−2.575, 0.363)	0.139
CpG4	61.78 ± 7.49	63.68 ± 2.88	0.323	−0.029 (−0.117, 0.059)	0.520	−2.664 (−5.118, 0.210)	**0.034**

*P* was adjusted by logistic regression for EH, age, BUA, smoking, drinking, BMI, ALP, TG, HDL, LDL, and Hcy. ^*∗*^ *P* was calculated by multiple linear regression model including gender, age, BUA, smoking, drinking, BMI, ALP, TG, HDL, LDL, and Hcy. BMI: body mass index; ALP: alkaline phosphatase; TG: triglycerides; HDL: high-density lipoprotein; LDL: low-density lipoprotein; Hcy: homocysteine.
